# Traumatic Stricture of Ten Years’ Standing, Complicated with Fistula Perinei: Urethrotomy

**Published:** 1858-12

**Authors:** Ellery P. Smith

**Affiliations:** Buffalo, New York


ART. II.—*Traumatic Stricture of Ten Years' Standing, complicated with Fistula Perinæi; Urethrotomy.* By ELLERY P. SMITH, M. D., Buffalo, New York.

I was called to examine the case of H. B., æt. 40, September 7th, 1858. The history of the case is as follows:

Ten years ago he was accidentally injured about the pelvis by machinery, being caught and badly crushed between large iron wheels. He passed some blood by the urethra, and had retention of urine, for which the catheter was made use of a number of times. Three years subsequently, he could only pass water in a very small stream, and for the past three years it has escaped only in drops. In the latter part of May last, perineal abscess formed and opened, and since that time all the urine, with the exception of a small spoonful or two daily, has passed through two fistulous openings in the perinæum. The man's general health is tolerably good, although there is considerable emaciation. Pulse 80; tongue clean; the bowels are habitually constipated. He complains of a constant severe pain in the perinæum, and has an orchitis of the right testicle, evidently the result of irritation. The prostate gland is much enlarged, but not tender on pressure. The passing of a No. 8 silver catheter caused great pain, and on reaching the membranous portion of the urethra the presence of a firm, narrow stricture, was at once detected, through which the instrument would not pass. I was enabled, however, after long and patient manipulation, chloroform having been administered, to pass a No. 3 steel sound about half an inch into the stricture, but the natural relations of the parts had been so changed by infiltration and inflammation, that I found it impossible to reach the bladder with that or any other sized instrument. The patient being exceedingly anxious for relief, and much discouraged with his present condition, I advised the external division of the stricture, to which he consented.

On Friday, Sept. 10th, assisted by Drs. Winne, Loomis, Hauenstien, and Brown, the patient having been placed in the position for lithotomy, and firmly secured, chloroform was administered, and a No. 3 steel staff, grooved on its under surface, introduced, and engaged for about half an inch in the stricture. I then made an incision, one and a-half inches long, in the median line of the perinæum, to within three-fourths of an inch of the anus; the transverse perinæl artery was divided and required a ligature. A careful dissection enabled me to reach the bulb, which was divided with but trifling hæmorrhage, and the staff reached; I then cautiously divided the stricture, from before backwards—this division extended altogether about three-fourths of an inch — and was attended with some difficulty, owing to the depth of the perinæum and the oozing of blood. The staff being withdrawn, I passed a No. 8 catheter down to the divided part, and after considerable trouble, succeeded in lodging it firmly in the contracted neck of the bladder, urine escaping through it. The catheter was secured in the usual way with tapes, and the wound in the perinæum slightly drawn together with a single suture.

℞ Morph. Sulph, gr. i.

Sept. 11. Saw the patient at 10, A. M. He has passed a good night, and feels bright and cheerful. Pulse 80; no fever. Over one pint of urine has escaped through the catheter.

Sept. 12. Last night had some pain in the region of the bladder, which yielded at once to anodynes and warm fomentations; has passed during the last twenty-four hours, nearly two quarts of urine through catheter, and but a trifling quantity through the wound in the perinæum.

Sept. 14. Continues improving. Makes water through catheter in a small stream; wound in perinæum cicatrizing.

Sept. 18. Removed the catheter, and reïntroduced it again without difficulty. Wound in perinæum entirely closed.

Oct. 10. The man is nearly well; has improved greatly in flesh and general health. Every third day he introduces the instrument, for six or eight hours, without difficulty.

Oct. 25th. Continued improvement, in fact he is quite well, is able to retain urine for five or six hours, and passes it in a full stream. I consider the termination of this case somewhat remarkable, from the fact that at no time since the operation has a bad symptom been present, not even the febrile reäction usually attending an operation of such magnitude.

